# Deconstructing knowledge brokering for commissioned rapid reviews: an observational study

**DOI:** 10.1186/s12961-018-0389-7

**Published:** 2018-12-12

**Authors:** G. Moore, S. Redman, P. Butow, A. Haynes

**Affiliations:** 1 The Sax Institute, PO Box K617, Haymarket NSW 1240, Ultimo, NSW 2007 Australia; 2 School of Public Health, Sydney Medical School, The University of Sydney, Edward Ford Building (A27), Sydney, NSW 2006 Australia; 3 Centre for Medical Psychology & Evidence-based Decision-making (CeMPED), Level 6 North, The Lifehouse, The University of Sydney, 119-143 Missenden Rd (C39Z), Sydney, NSW 2006 Australia

**Keywords:** knowledge broker, knowledge brokering, rapid synthesis, rapid review, health policy, research utilisation

## Abstract

**Background:**

Knowledge brokers are increasingly used by policy agencies, yet little is known about how they engage with policy-makers and facilitate discussions with them about their research needs. This study examines knowledge brokers’ behaviour in one-off interactions with policy-makers commissioning rapid reviews. It describes how knowledge brokers engage with policy-makers, build trust and gain agreement about the review’s parameters.

**Methods:**

We observed and transcribed 15 structured knowledge brokering sessions and used line-by-line analysis to derive, test and refine a coding schedule. The final coding schedule was applied to all transcripts. We assigned 35 codes to three tasks identified in the data, namely eliciting information, exploring the policy context and negotiating the content of reviews.

**Results:**

The knowledge brokers we observed were skilled facilitators who built trust by their open stance, neutrality, and knowledge of research and policy contexts. Trust engendered an interplay of expertise in which review questions and scope were clarified and contextual factors evaluated. Negotiation about the content of the review focused on understanding how it would contribute to the policy process, comparing options and assessing feasibility. Key functions of knowledge brokers included eliciting and clarifying information, linking the review questions to the context and purpose, moving fluidly between policy and research perspectives, and weighing up review options against policy objectives. Four knowledge brokering roles were identified, namely diagnostic, facilitative, deliberative and interpretative.

**Conclusions:**

This study identified ways in which knowledge brokers established rapport with policy-makers who commissioned reviews, enabled disclosure of essential information and explored contextual factors that affected the review’s purpose and intended use. Knowledge brokers were competent in the discourse and conventions of both policy and research and were skilled in negotiating complex policy and political environments, assisting policy-makers to evaluate options and craft a review proposal that was targeted, responsive and feasible. Mutuality, respect and an interplay of expertise were integral to the knowledge brokering process. Future research might usefully examine whether other rapid review programmes using knowledge brokers have similar results as well as the transferability of the four knowledge brokering roles to other contexts and settings.

**Electronic supplementary material:**

The online version of this article (10.1186/s12961-018-0389-7) contains supplementary material, which is available to authorized users.

## Introduction

There is now substantial literature documenting the barriers encountered by policy-makers who seek to access and use research in decision-making [1, 2]. A number of strategies have been posited to address these barriers, including increasing access to timely, relevant research [1, 3–6], building capacity to access and use research [7–9], interaction between policy-makers and researchers [10–13], and coproduction, in which both policy-makers and researchers jointly engage in the design, implementation and interpretation of research [14, 15]. One strategy that is gaining currency is the use of knowledge brokers. Initially described in broad terms [16], knowledge brokers’ roles are commonly aligned with one of three main types of activity. These are knowledge management, where knowledge brokers develop systems and processes to access and disseminate research [17–19]; linkage and exchange, where they facilitate interaction between policy-makers and researchers [17, 20, 21]; and capacity-building, where they provide individualised training and one-to-one support [18, 22–26].

More recently, there have been attempts to articulate and differentiate the ways in which knowledge brokers are used. For example, Dobbins et al. [22], among others, described knowledge brokers’ roles as conducting needs assessments, scanning the horizon, building capacity and supporting organisational change. Dagenais et al. [18] pointed to the importance of brokers’ interaction with policy-makers and programme managers through formal and informal networks and their role in relaying information and new knowledge.

Many of these roles seem to be based on an assumption that knowledge brokers address a gap in policy-makers’ ability to find and use research, for example, because individual policy-makers have insufficient skills or their organisations lack capacity or because policy-makers and researchers cannot communicate in a meaningful way. However, this assumption is now being challenged. Indeed, many policy-makers have a sophisticated understanding of research [27] or are themselves researchers and, while policy agency capacity for research may be limited in some instances, there are a number of models emerging which address this in considered ways [7, 28].

This shift in emphasis points to a different kind of knowledge brokering role that aligns more closely with notions of shared expertise than with deficit models. Conklin et al. [17] reported their work in creating relationships, promoting mutual understanding, and facilitating exchange across social and cultural boundaries. As ‘one-among’ policy-makers and researchers and with a conceptual capacity to move freely within both, knowledge brokers can engage at a deeper level than is typical of other facilitative approaches. They are therefore uniquely positioned to work with policy-makers rather than stand between them and researchers, straddling a divide [21].

When knowledge brokers have an in-depth understanding of the policy domain, they can engage with the complexity inherent in policy processes where interests, ideologies, alliances and stakeholders compete [29, 30]. Indeed, policy processes are often contested and knowledge brokers must be sensitive to the, at times, fraught issues, be able to establish trust, and work in a way that is respectful of these circumstances. Knowledge brokers’ activities are therefore highly context dependent. The skills that are required in one context are likely to differ significantly from those needed in another and include facilitating partnerships, clarifying research needs or supporting organisational change. Therefore, an analysis of knowledge brokers’ activities needs to be conscious of, and reflect, the ‘real world’ environments in which they occur.

To date, little information is available about knowledge brokers’ practices or the mechanisms by which they work [25, 31]. This study therefore aimed to explore in depth the processes used by knowledge brokers and to shed light on their practices and the ways in which they work, thus drawing conclusions that may be broadly applicable to other knowledge brokers using similar models. To achieve this, we employed a case study approach and examined the operation of knowledge brokers in the New South Wales Evidence Check programme, which supports policy-makers to commission rapid reviews. In this programme, knowledge brokers work with policy-makers to specify the requirements of their review and draw up a rapid review proposal that will inform the contracted researchers about the scope of work required.

While there is little evidence of the effectiveness of knowledge brokers overall [32, 33], knowledge brokers in the Evidence Check programme have been found to make a significant difference to the clarity of review proposals from the perspective of representative reviewers, that is, previous authors of Evidence Check rapid reviews commissioned using knowledge brokers. Using a sample of 60 rapid reviews, representative reviewers were asked to assess the clarity of review proposals written before (*n* = 60) and after (*n* = 60) knowledge brokering while blind to the before and after status of each proposal. Clarity pertained to the policy problem, the review questions, scope, methods and report format. Representative reviewers were also asked about their confidence that they could meet policy-makers’ needs based on the information contained in the proposal. The proposals were scored using a 6-point Likert scale and the scores for all six questions showed significant improvements following knowledge brokering [34]. Moreover, a recent study examining whether rapid reviews commissioned using Evidence Check were useful for policy-makers found that 89% of rapid reviews had been used by the agencies who commissioned them, in multiple and diverse ways [35]. More detailed information about the Evidence Check programme is available elsewhere [36].

In the Evidence Check programme, a knowledge broker meets with the commissioning policy-maker(s) to understand why the review is being commissioned and how it will be used, and to agree on the scope and parameters of the review, including what is feasible within the time frame and budget. The knowledge broker has to translate these parameters in a way that will be understandable to researchers and without divulging any confidential information provided during the discussion with the policy-makers. This study focuses on this complex interpretative process.

The specific objectives of this study were to analyse the ways in which knowledge brokers (1) elicit and clarify information, (2) understand the context in which the review will be used, and (3) gain agreement on the review’s final content.

## Methods

### Enrolment in the study

All policy teams who commissioned a rapid review from the Sax Institute were invited to participate in the study. Enrolment continued until 15 policy teams were enrolled. A sample size of 15 was chosen based on Guest et al.’s proposal [37] that 12 interviews are likely to achieve a representative sample.

Three policy agencies declined to participate in relation to three knowledge brokering sessions due to the sensitivity of the subject matter; all participants provided informed consent. The first knowledge brokering session was held in April 2014 and the last in July 2015.

Sessions were included in the study consecutively from the commencement date. Independent of the research process, knowledge brokers were allocated to sessions according to their availability. No other criteria were used; however, two Evidence Checks were judged to be of a level of complexity that required the input of more experienced brokers, thus specific brokers were approached to manage these Evidence Checks in accordance with the programme’s usual protocols.

### Data collection

The knowledge brokering sessions that formed the basis of this study were conducted face to face, with some members participating via teleconference, if needed. Policy agencies were represented at sessions by at least one senior staff member with responsibility for implementing review findings and who could therefore make decisions about the review’s content. One of the Sax Institute project managers, who coordinate the review process, was assigned to each review and attended the knowledge brokering session.

The lead author (GM) attended all sessions and made and transcribed audio recordings. Knowledge brokers and policy participants completed a standard post-session form developed and administered as part of the study (Additional file 1), focusing on changes in understanding about the policy problem and about the way in which the review questions were specified. Participants were asked about the perceived value of the session and were invited to add any other comments. All data were de-identified. To further de-identify knowledge brokers and participants, we have sometimes altered the names of agencies or the gender or numbers of participants in the examples taken from the transcripts.

After the sessions, policy participants and knowledge brokers were contacted up to three times and invited to complete a survey. The survey included two free text descriptions of their perceptions of what had changed as a result of the knowledge brokering session and any additional comments. Five questions using a 4-point Likert scale examined the degree to which respondents’ reported changes to their understanding of the policy problem, changes to the review questions, and the perceived importance of these changes. Respondents were also asked to what degree information needed to tailor the review to policy-makers’ needs had been elicited in the session.

### Analysis

GM analysed two transcripts using Charmaz’s grounded theory approach [38] with word-by-word and line-by-line analysis as an orientation to the text of the transcripts and to ensure data coding was based on the data alone. The results of the analysis were used to develop a first coding schedule of 38 codes, which was reviewed by two authors (SR and PB). The codes were assigned to one of the three knowledge brokering tasks identified in the data, as eliciting information, exploring the policy context, and negotiating the content of reviews; this structure was used to report the results.

The schedule was applied to two new transcripts, the results were reviewed by SR and PB, and the schedule was subsequently amended. This second schedule of 15 codes was applied to five transcripts using NVIVO 11 and 21 new codes were identified. The third and final coding schedule of 36 codes was then applied to all transcripts until thematic saturation was reached at the ninth transcript and tested with the tenth; no new themes were identified. GM prepared draft findings based on the 10 transcripts and the final five transcripts were coded with a view to testing and amplifying the findings. Two codes were merged during the analysis resulting in a final set of 35 codes. GM synthesised the data from the transcripts and the findings were then reviewed by all authors.

Early analysis suggested that variation in session outcomes was affected by differences in brokers’ levels of knowledge brokering experience. Consequently, we assigned knowledge brokers to one of three categories according to their level of experience at the commencement of the study – those who had conducted 1–10 sessions, 11–20 sessions, or 21 or more sessions were designated ‘less experienced’, ‘experienced’ and ‘very experienced’, respectively. NVIVO 11 was used to collate codes occurring across broker level of experience to explore commonalities and differences between their approaches. We also sought to detail their specific activities in the knowledge brokering sessions.

To assess the experience of policy participants in knowledge brokering sessions, we selected the ‘policy liaison person’ for each review to enable a comparison between their experience and that of the knowledge brokers. The policy liaison persons were not necessarily the most senior policy-makers at a knowledge brokering session; however, they liaised directly with the Sax Institute and therefore received preliminary information about the roles of policy participants and knowledge brokers, the process of proposal development, and the conduct and publication of the review.

We defined policy participants as having ‘some experience’ if they had attended one or more knowledge brokering sessions prior to the study, or as having ‘no experience’ if they had not attended a session prior to the study.

## Results

### Study sample

#### Reviews and agencies

We observed and analysed the interactions of 15 knowledge brokering sessions concerning 15 Evidence Check rapid reviews commissioned by six agencies, including two large agencies with multiple divisions. One agency commissioned five reviews, one commissioned four reviews, two agencies each commissioned two reviews, and two agencies each commissioned one review (Table 1).

**Table 1:** Distribution of sessions by type of agency

Agency type	No. sessions
Regional statutory health authority 1	2
Regional statutory health authority 2	5
Regional statutory health authority 3	2
National non-government agency	1
Regional government agency (health)	4
National government agency (health)	1
	15

#### Sessions

Of the 15 sessions (one per review), 12 were held at the policy agency and three at the Sax Institute. All sessions were conducted face to face, with one person participating via teleconference in each of three sessions. The average duration was 66 min, ranging from 42 to 120 min.

#### Session participants

##### Knowledge brokers

Five knowledge brokers conducted the sessions included in this study. Of these, two were very experienced (Brokers 1 and 3), one was experienced (Broker 5), and two were less experienced (Brokers 2 and 4; Table 2). Four knowledge brokers were female and one was male. Four knowledge brokers held substantive research positions and one held a senior management position. Coding per broker level of experience is reported in Table 3.

**Table 2:** Number of sessions conducted by knowledge brokers before and during the study

	No. sessions conducted before the study	No. sessions conducted during the study	Coded in the study
Knowledge broker 1	32	7	Very experienced
Knowledge broker 3	21	4	Very experienced
Knowledge broker 5	11	1	Experienced
Knowledge broker 2	3	2	Less experienced
Knowledge broker 4	0	1	Less experienced

**Table 3:** Knowledge broker behaviours coded by level of experience

	Knowledge broker behaviours	Very experienced brokers	Experienced brokers	Less experienced brokers	No consistent pattern
1	Asking closed questions	Yes	Yes	Yes	
2	Asking open ended questions	Yes	Yes	Yes	
3	Asking more detail	Yes	Yes	Yes	
4	Building trust	Yes	Yes	Yes	
5	Checking understanding	Yes	Yes	Yes	
6	Managing the session	Yes	Yes	Yes	
7	Diagnostics	Yes	Yes	Yes	
8	Gaining agreement	Yes	Yes	Yes	
9	Managing interludes	Yes	Yes	Yes	
10	Letting them talk	Yes	Yes	Yes	
11	Open dialogue	Yes	Yes	Yes	
12	Pointing to problems or solutions	Yes	Yes	Yes	
13	Positive regard	Yes	Yes	Yes	
14	Referencing the reviewer	Yes	Yes	Yes	
15	Reflecting back	Yes	Yes	Yes	
16	Filtering	Yes	Yes		
17	Frank disclosure	Yes	Yes		
18	State re-state	Yes	Yes		
19	Understanding context	Yes	Yes		
20	Using humour	Yes	Yes		
21	Negotiating	Yes	Yes		
22	Setting expectations	Yes	Yes		
23	Explaining	Yes	Yes		
24	Power or ownership	Yes	Yes		
25	Being neutral	Yes			
26	Personal anecdote	Yes			
27	Combining expertise	Yes			
28	Frame-re-frame	Yes			
29	Interplay of expertise	Yes			
30	Highlighting inconsistencies				Yes
31	Accord and discord				Yes
32	Adding value				Yes
33	Testing constructs				Yes
34	No trust				Yes
35	Using examples				Yes

##### Policy participants

There were 53 policy participants in the study. The average number of policy participants at a session was 3.5, ranging from 1 to 7. Seven participants were male and 46 were female. Fifteen policy participants were nominated as liaison persons in the study. One was nominated as policy liaison person for two reviews; this person has been counted once and the denominator reduced to 14 persons. Two policy liaison persons were male (14%) and 12 (86%) were female (Table 4). Further, three liaison persons (21%) had some experience of a knowledge brokering session prior to the study. However, some participants attended more than one session during the study period and therefore gained experience as they progressed. Two liaison persons (14%) attended two sessions during the study and one person (7%) attended three sessions during the study.

**Table 4:** Number of sessions attended by policy liaison people before and during the study

	No. sessions attended before the study	No. sessions attended during the study (in any role)	Coded in the study
Policy liaison person 1	0	1	No experience
Policy liaison person 2^a^	1	3	Some experience
Policy liaison person 3	0	1	No experience
Policy liaison person 4	0	1	No experience
Policy liaison person 5	0	1	No experience
Policy liaison person 6^a^	1	3	Some experience
Policy liaison person 7	0	2	No experience
Policy liaison person 8	0	1	No experience
Policy liaison person 9	0	1	No experience
Policy liaison person 10	0	1	No experience
Policy liaison person 11	0	1	No experience
Policy liaison person 12	0	2	No experience
Policy liaison person 13	1	1	Some experience
Policy liaison person 14	0	1	No experience
Policy liaison person 15	1	1	Some experience

^a^Policy liaison persons 2 and 6 are the same person who has been counted once

### Responses to the survey questions

The response rate for the knowledge broker survey was 65% and for the policy participants 26%; therefore, the results must be considered with caution (Tables 5 and 6).

**Table 5:** Knowledge brokers' responses to survey questions

KBS^a^ No.	Did the KBS change your understanding of the problem?				Was this change in understanding important?				Did the KBS change the way the research question was specified?				Was this change in research question important?				Was the information needed to target the review to the policy-makers’ needs elicited in the session?				Overall, did you find the KBS valuable?			
	Question 2				Question 3				Question 4				Question 5				Question 6				Question 7			
	Yes, a lot	Yes, a little	No, not at all	Don’t know	Yes, a lot	Yes, a little	No, not at all	Don’t know	Yes, a lot	Yes, a little	No, not at all	Don’t know	Yes, a lot	Yes, a little	No, not at all	Don’t know	Yes, a lot	Yes, a little	No, not at all	Don’t know	Yes, a lot	Yes, a little	No, not at all	Don’t know
1	0	1	0	0	1	0	0	0	1	0	0	0	1	0	0	0	1	0	0	0	1	0	0	0
2	0	1	0	0	1	0	0	0	0	1	0	0	0	1	0	0	1	0	0	0	1	0	0	0
5	0	1	0	0	1	0	0	0	0	1	0	0	0	1	0	0	1	0	0	0	1	0	0	0
6	0	0	1	0	1	0	0	0	1	0	0	0	1	0	0	0	0	0	1	0	0	0	1	0
7a^b^	1	0	0	0	1	0	0	0	1	0	0	0	1	0	0	0	1	0	0	0	1	0	0	0
7b^b^	1	0	0	0	1	0	0	0	0	1	0	0	1	0	0	0	1	0	0	0	1	0	0	0
7c^b^	1	0	0	0	1	0	0	0	0	1	0	0	1	0	0	0	1	0	0	0	1	0	0	0
8	0	1	0	0	0	1	0	0	0	1	0	0	0	1	0	0	0	1	0	0	1	0	0	0
9	1	0	0	0	1	0	0	0	0	1	0	0	0	1	0	0	1	0	0	0	1	0	0	0
10	0	1	0	0	0	1	0	0	0	1	0	0	0	1	0	0	0	1	0	0	0	1	0	0
14	1	0	0	0	1	0	0	0	1	0	0	0	1	0	0	0	0	1	0	0	1	0	0	0
n	5	5	1	0	9	2	0	0	4	7	0	0	6	5	0	0	7	3	1	0	9	1	1	0
%	45	45	9	0	82	18	0	0	36	64	0	0	55	45	0	0	64	27	9	0	82	9	9	0

^a^*KBS* knowledge brokering session

^b^In this session, three related rapid reviews were knowledge brokered and the broker provided responses for each one

**Table 6:** Policy-makers’ responses to the survey questions

KBS^a^ No.	Policy participant no.	Did the KBS change your understanding of the problem?				Was this change in understanding important?				Did the KBS change the way the research question was specified?				Was this change in research question important?				Was the information needed to target the review to the policy-makers’ needs elicited in the session?				Overall, did you find the KBS valuable?			
		Question 2				Question 3				Question 4				Question 5				Question 6				Question 7			
		Yes, a lot	Yes, a little	No, not at all	Don’t know	Yes, a lot	Yes, a little	No, not at all	Don’t know	Yes, a lot	Yes, a little	No, not at all	Don’t know	Yes, a lot	Yes, a little	No, not at all	Don’t know	Yes, a lot	Yes, a little	No, not at all	Don’t know	Yes, a lot	Yes, a little	No, not at all	Don’t know
1	1	0	1	0	0	0	1	0	0	0	1	0	0	0	1	0	0	1	0	0	0	1	0	0	0
1	2	0	1	0	0	0	1	0	0	0	0	1	0	0	0	0	0	1	0	0	0	1	0	0	0
3	7	0	1	0	0	0	1	0	0	0	1	0	0	0	1	0	0	0	1	0	0	0	1	0	0
3	11	0	0	0	1	0	0	0	1	0	1	0	0	0	1	0	0	0	1	0	0	0	1	0	0
4	12	0	0	1	0	0	0	0	0	0	1	0	0	1	0	0	0	1	0	0	0	1	0	0	0
4	13	0	0	1	0	0	0	1	0	0	1	0	0	0	0	0	1	1	0	0	0	1	0	0	0
5	15	0	1	0	0	0	1	0	0	0	1	0	0	0	1	0	0	0	1	0	0	0	1	0	0
6	19	0	0	1	0	0	0	0	0	1	0	0	0	1	0	0	0	0	0	0	1	0	0	0	1
7	24	0	0	1	0	0	0	0	0	0	1	0	0	0	1	0	0	0	1	0	0	1	0	0	0
8	30	0	0	1	0	0	0	1	0	1	0	0	0	0	1	0	0	0	1	0	0	1	0	0	0
8	32	0	1	0	0	0	1	0	0	0	1	0	0	1	0	0	0	1	0	0	0	1	0	0	0
9	33	0	0	1	0	0	0	1	0	0	1	0	0	0	1	0	0	1	0	0	0	1	0	0	0
9	34	0	1	0	0	0	1	0	0	0	1	0	0	1	0	0	0	1	0	0	0	1	0	0	0
	n	0	6	6	1	0	6	3	1	2	10	1	0	4	7	0	1	7	5	0	1	9	3	0	1
	%	0	46	46	8	0	46	23	8	15	77	8	0	31	54	0	8	54	38	0	8	69	23	0	8

^a^*KBS* knowledge brokering session

Overall, there was agreement among the knowledge broker and policy respondents about changes to the review questions (100% and 91%, respectively) and the importance of these changes (100% and 85%, respectively). Most knowledge brokers and policy-makers said that the information needed to tailor the review to policy-makers’ needs was elicited during the session (91% and 92%, respectively). Almost all knowledge brokers and policy participants (91% and 92%, respectively) said they found the knowledge brokering session valuable or very valuable. Understandably, knowledge brokers and policy respondents differed in the degree to which their understanding of the policy problem changed during the knowledge brokering session (90% and 46%, respectively) and the importance of these changes (100% and 46%, respectively). Further, 46% of policy respondents said their understanding of the policy problem had not changed, compared to 9% of knowledge brokers.

To highlight similarities and differences in knowledge broker and policy-maker responses, we provide examples taken from three knowledge brokering sessions (Box 1).

### Identification of knowledge broker tasks

Working inductively from our analysis of the data, we identified three key tasks for knowledge brokers, namely (1) eliciting and clarifying information, (2) exploring the underlying contextual issues and (3) gaining agreement about the content of the review. The kinds of activities knowledge brokers used in enacting these tasks are summarised in Box 2. Across these activities, we found that brokers enacted roles that were facilitative, diagnostic, interpretative or deliberative (Table 7). While more than one role may have been operational at different points during the sessions, we found that they played out roughly in the order in which we have described them, i.e. in the early parts of a session brokers used mostly their facilitative and diagnostic roles and in the later parts of a session, they moved more into their interpretative and deliberative roles.

**Table 7:** Knowledge broker roles

Facilitative role	Diagnostic role	Interpretative role	Deliberative role
• Establishing and maintaining trust and creating an open dialogue • Facilitating understanding across social and cultural boundaries • Eliciting disclosure of sensitive information and gaining greater specificity	• Clarifying research needs for a specific policy process • Identifying contextual factors that may influence research use • Refining research questions and scope to reflect purpose and context	• Moving between and aligning research and policy perspectives • Communicating in the language and conventions of both policy and research domains • Conveying what is meant and intended in one domain so it can be understood and enacted in the other	• Weighing up research options in terms of their contribution to a policy objective • Surfacing inconsistencies or conflicting viewpoints for resolution • Fostering judgements that enable action and decision-making going forward

### Task 1: Establishing trust and eliciting information

Mutual trust and respect have been acknowledged as critical in establishing and sustaining genuine partnerships [14, 39]. Yet, knowledge brokers and policy-makers in this study had little time available for the kinds of extended interaction that enable a recognition of the strengths and expertise in the other. Further, when trust was not formed, knowledge brokers found it difficult to elicit the kinds of confidential contextual information that would enable them to fully understand how the findings would contribute to a particular process alongside other inputs to the policy process. Without this understanding, the broker was less able to provide the needed information for the researcher who would undertake the review. Trust, therefore, needed to be established quite quickly at the commencement of the session.

#### Creating an open dialogue

In their facilitative role, knowledge brokers’ initial objective appeared to be to create conditions where an open dialogue could take place and a sense of safety in which the policy team could speak freely about their policy context and the issues at hand.

Brokers differed in the way they engaged, and later disengaged, with policy-makers; however, there were common elements in the content of brokers’ opening discourse. They acknowledged policy-makers’ work in preparing the commissioning tool (the form that policy-makers complete describing what they want from a rapid review) as a starting point for the discussion. Most brokers pointed to their own experience as a broker or as a researcher and to their preparation for the session, such as reading background information about the agency and the review topic, scanning the literature and thinking through the review questions. They affirmed their responsiveness to policy-makers’ needs in ensuring the review would be fit for purpose. Experienced brokers would communicate their neutrality in terms of the content of the review:

Broker 5: “*My job is not to tell you to do A versus B, I am absolutely agnostic about that. My job is to help you to get what you want out of this evidence review, and basically to make sure that when I write those two, three or four pages for the researchers to look at, they understand what it is that you want*.”

Others openly acknowledged the uncertainty policy-makers often felt at the beginning of the session and described the shared nature of that experience. This mutuality was characteristic of all sessions and would be an essential aspect throughout the brokering process.

Broker 1: “*Let me just assure you that at the end of this my brain hurts as much as yours and I think the tag line for this is that there is no such thing as a dumb question or a dumb answer!*”

Control of the session varied across participant groups and could devolve during the session. Experienced brokers who took an overt leadership role found it easier to manage the progression of the session, but some policy-makers assumed control. This may relate to the policy-maker’s perception about the sensitivity of the subject matter, to a real or perceived lack of skill on the part of the knowledge broker, or to other factors such as the seniority or personality of the policy-maker. Some discomfort about who was directing the discussion was common at the beginning of most sessions and the more experienced brokers managed this by addressing it explicitly, negotiating temporary leadership.

This power transaction seemed intricately linked to the establishment of trust, which was not a given, but had to be won in the early exchange. On a couple of occasions, policy-makers asserted their ownership of the content of the review – seeking explicit control of the session may have been a function of their uncertainty about the process or perceived loss of authority as the brokers took leadership. Therefore, brokers often opened with clear statements about their role in guiding the session and were explicit about policy-makers’ authority in terms of the review’s content.

Broker 3: “*Nothing goes anywhere until you have approved it. Then the Sax team does their part and tries to attract the right person for the job and you get a say in that as well. So, things don’t happen without your consent*.”

#### Understanding the purpose of the review

One of the ways brokers gained trust was by signalling their familiarity with the policy context relevant to the review in question, and they did this in often quite sophisticated and nuanced ways. One experienced broker touched on the extent of her knowledge by referencing a national guideline on an agency’s website that had been recently rescinded and pointing to a parallel change in that agency’s policy direction. Another broker referenced a conceptual framework frequently utilised in the literature on the specified topic, using its components to structure her questions to the policy team; they immediately recognised and responded to the issues she raised. In all but three of the sessions, the brokers pointed to their knowledge of the policy context and of the issues likely to occur in a particular health system or policy process.

The shared recognition of the complexity of the policy context enabled the dialogue to move beyond the policy’s development to the underlying issues that might influence its direction. In their diagnostic hat, experienced brokers worked to unravel and understand these underlying factors and establish their relationship to the proposed review. This kind of deeper exploration only occurred when trust was established in the early interaction; indeed, it appeared that sensitive information was often revealed precisely at the point at which policy-makers determined that the broker could be trusted.

In one session, a policy-maker was describing the factors that contributed to staff in emergency departments failing to implement a policy directive when a relatively rare clinical event occurred. As she described the situation, she broke off and, turning to the broker, showed her discomfort in disclosing information which the broker might perceive in a negative light. The broker immediately indicated her understanding of the policy-maker’s dilemma, calling on an experience in which she herself had been vulnerable. The exchange, while brief, seemed quite personal, but once the broker had signalled her understanding of the circumstances, the policy-maker was able to continue her analysis of the causes of the policy’s failure.

Policy participant 4:* “I feel I need to say that it’s a highly sensitive topic –* [You] *do understand the sensitivity of what we are talking about*.”

Broker 1: “*Yes. As I was reading the policy directive, I remembered that as a very junior doctor working in* [an emergency department] *… you never forget it*.”

The broker’s capacity to respond empathically and non-judgementally both reassured the policy participant and facilitated the smooth progression of the session.

In some instances, less experienced brokers moved directly to clarifying the review questions without exploring the background to the review and the policy context or without establishing rapport. As a result, participants received no direction about the process or structure of the session and no guidance about the issues to be addressed. The brokers may have assumed that the information in the commissioning tool was sufficient or the value of the early exploration may not have been evident. In addition, participants themselves may have been keen to move to the more tangible questions which seemed to address their policy questions in more concrete ways. Without a clear understanding about how the review would be used, brokers found it difficult to negotiate what would be in or out of the review, and there was no central focus against which the relevance of options could be evaluated.

In one session, the policy team sought evidence about prevention in two disease groups with very different trajectories and bases in the evidence; with no exploration about the policy background, the underlying differences between the two main questions were never made explicit. The broker’s questions elicited contradictory responses and participants were unable to agree on the review questions and scope; the broker was unable to reconcile these inconsistencies. Because the background to the review was not discussed and the differences in the two approaches to prevention were not explored, assessing the extent to which both could be encompassed in a single review became difficult. The broker addressed this to a degree in the scope, creating sub-questions relevant to each specialty; however, without any clear delineation, the review questions became complex and unwieldy.

#### When sensitive information is withheld

When sensitive information that could have progressed understanding was not disclosed, brokers were left to infer the focus for the review, based on background information provided by the policy-makers in advance of the session and on their own knowledge of the policy process.

In one review, there was little interaction at the beginning of the session and little rapport among the policy participants, many of whom had not met before. In the opening minutes of the session, a senior clinician who was part of the commissioning team asked the lead policy-maker whether she could talk about the adverse events that triggered the review. The policy-maker said she could not.

The knowledge broker accepted that full information about the problem would not be provided but struggled to reconcile the multiple viewpoints among the policy team about what was wanted from the review and she had limited ability to define the research question and the scope of the review appropriately. Consequently, the broker drafted a review proposal based on the commissioning tool with little information about the actual needs of the policy team.

An absence of interaction and rapport that was obvious at the beginning of the session may have accounted for this non-disclosure, at least in part. It may have reflected a lack of skill on the part of a less experienced broker in facilitating trust, or the lack of established relationships among the policy participants, some of whom were meeting for the first time; however, it may also have reflected the lead policy-maker’s concern about tabling for discussion an adverse event not yet in the public domain. Nevertheless, in most instances, policy-makers readily disclosed sensitive information, allowing the broker to quickly become cognisant of the complexities underpinning the review.

### Task 2: Clarifying research needs and understanding context

One of the most important and complex tasks for knowledge brokers was to understand the perspective from which the policy-maker viewed the policy problem. The concepts and linguistic constructs used by policy-makers seemed to differ quite radically at times from those of researchers; that is, the same word could have a particular and shared meaning for policy-makers and have multiple and diverse meanings for researchers. Therefore, for the knowledge brokers, understanding exactly what a policy team wanted the researchers to focus on required careful exploration. Implicit in the dialogue was the movement by the knowledge broker to and from policy and research perspectives until what was needed was clearly defined and could be conveyed to the researchers who would conduct the review. This translational capacity was characteristic of experienced knowledge brokers. To begin, however, brokers drew on their diagnostic role.

#### Exploring contextual factors influencing the review

Most knowledge brokers began by eliciting information about the key events that led to the commissioning of the review, using broad statements that allowed policy-makers to engage in the process and provide information about the policy’s background.

Broker 5: “*I think a good way to start would be for you to tell me a little bit about why we are here. What prompted this request? Why now? Because the first thing I am going to do is write a few paragraphs explaining a little bit about the background and context, for the researchers*.”

Policy-makers were on home ground here and could readily provide the kinds of descriptive detail that would help brokers understand what was required and would align the review to the policy context; however, some policy-makers were unsure about exactly what information the broker needed. Brokers used open-ended questions to explore some of the key issues, such as how the review would be positioned and the roles of stakeholders in the policy process.

Broker 3: “*So, one of the things I noticed about the commissioning tool is that you are looking for a really practical piece of work that will be used in the real world. Who are these ‘real-world’ people that you had in mind?*”

Policy-makers’ narratives were sometimes dense and multi-layered; in describing the context in which a particular policy decision was unfolding, information was disclosed that was not pertinent to the review’s content or direction. Brokers therefore needed to filter the information, allowing what was disclosed to stand, but bringing the discussion back to the review’s main focus. This could be a delicate task as brokers responded to subtle cues, drawing out what was implicit in what was being said, so that its importance in a particular process could be explored and evaluated:

Broker 3: “*Now, one of the things I was intrigued about, it says* [in the commissioning tool] *‘there is a draft policy developed but not progressed’. So, I was wondering whether you could sort of fill me in on that? What’s going on there and why it hasn’t progressed? It could be just some common-sense reason like lack of time, but there could be other things as well*.”

When needed, knowledge brokers prompted policy-makers to provide more detail, until they were comfortable they had a clear understanding of what was required and how the review findings would be used in the particular policy under discussion.

#### Evaluating contextual information

Contested policy environments were a common underlying concern that policy-makers were sometimes hesitant to disclose. Brokers had to find a way to explore sensitive political issues and determine their relationship to the review in order to clarify the review questions and scope. One participant expressed the difficulty of negotiating an outcome in a fraught political environment.

Policy participant 19: “*This is a multi-stakeholder policy and there are a lot of very strong supporters. It’s going to be very tricky in terms of who is seen to have gotten their own way at the end of it*.”

One review looked at a multi-agency response to the management of an issue involving policy agencies, community services, specialist clinicians and hospital providers. Because the agency facilitating the policy’s development was a key player in this response, the policy-makers were keen to establish their impartiality and avoid a perception of bias towards a particular solution. Further, while the heads of agencies supported a joint response, there was disagreement among the policy participants about how this should play out in practice. The review question reflected something of this ambiguity and the broker needed to clarify the premise on which the question was based.

Broker 3: “*Is there an acceptance that the response is always going to be multi-agency?*”

Policy participant 4: “*No, but that’s what we support*.”

After further discussion, the broker included some of the contested issues in the scope of the review, asking the reviewers to highlight contextual information that would help policy-makers assess the applicability of the findings, such as information about jurisdictions, agency roles, governance structures, and barriers and enablers to implementation. While the policy team’s concern about their perceived partiality was not in itself relevant to the review, the underlying controversial management issues operating in the background that gave rise to their concern, were both pertinent to and appropriate for consideration in a rapid review.

In their diagnostic role, understanding the underlying issues enabled brokers to focus the review questions and scope in a way that would be consistent with the agency’s goals. In one session on clinical variation, the broker proposed outcome measures that would allow the policy team to advocate for the needs of children in the adult clinical variation space, as one of the policy teams’ main concerns was to address the assumption that solutions to variation in the adult domain could automatically be applied in the paediatric domain. In taking this approach, the broker was also able to reflect her understanding of the policy-makers’ experience.

Broker 1: “*You’ve got to understand the particulars of clinical variation in the paediatric space and if necessary be a strong advocate. Instead of saying as is quite often the case ‘well that really worked well in adults, we will just make it a quarter size’*.”

In several sessions there was an unspoken expectation that research could play a role in resolving an impasse in the policy process. In one instance, the policy team wanted to pre-empt a situation where time would be lost in debate between the conflicting views of members of a steering committee about the policy’s direction, which the commissioning team predicted would be difficult to resolve. Commissioning a review that examined the evidence for the several issues that the policy would address could both establish the evidence base and set the agenda in a neutral way; however, when the questions were too many and varied to be addressed in a single review, the need for the evidence to inform the policy and provide a tactical solution meant that policy-makers had difficulty prioritising among review questions.

Exploring the contextual elements enabled the broker to identify the underlying obstacles and table them for consideration. In this instance, the policy participants were then able to work out a process that allowed them to draw on the committee’s expertise in a neutral way, and the session could then address the content of the review itself.

#### Defining the scope of the review

Once the knowledge brokers had sufficient information about the problem and policy context, they moved to a more diagnostic approach and, using a question and answer modality, tested the meaning of the proposed review questions, ruling material in or out of scope, constructing a research question set that was clear and delineated, all of which together made a cohesive whole. Wearing their diagnostic hat, brokers checked the alignment of the questions with the policy-makers’ opening narrative, refining the language and structure until the question set was determined. In this example, Broker 1 and Policy participant 38 were specifying the scope of the review.

Broker 1: “*Now, in terms of ambulatory care are we talking about primary care, GPs?*” Policy participant 38: “*No*.” Broker 1: “*Are we talking about patients?*” Policy participant 38: “*Yes.*” Broker 1: “*Are community services in your ambit?*” Policy participant 38: “*I don’t think so, no*.” Broker 1: “*So basically outpatients, ED, and admissions are the ones. Ok, alright, that’s very helpful.*”

A common difficulty was the definition of key terms. Brokers sought to identify the meaning of words and phrases that underpinned the conduct of a review, and rule aspects of these broader concepts in or out of scope. Examples included ‘model of care’, ‘complex systems’, ‘best practice’, ‘effectiveness’, ‘large-scale change’ and ‘chronic disease’. Other definitions focused on the specifics of populations and settings such as ‘home care’, ‘school-based’, ‘inpatient’ or ‘adolescent’. As one broker commented:

Broker 1: “*It’s interesting, the use of language. For example, what is ‘effective’ to one person, their idea of effectiveness, is different to another person. So, I have got to understand what you need*.”

#### Clarifying the intent of the question

Knowledge brokers seemed to find delineating the scope of work relatively straightforward once key terms were defined and other basic parameters of the review decided, such as range of publication dates or the countries from which literature should be drawn. Sometimes, however, understanding what was needed proved more elusive; policy-makers had a clear idea of what they wanted but could not communicate this in a way that the knowledge broker could convey to potential reviewers.

One review looked at the use of surveys to capture patient experience. The policy team described the review’s purpose and questions in the commissioning tool given to the knowledge broker ahead of the session. Their first question was “What are the best ways to assess patient experience?”

The question could be understood from a number of perspectives and each could be a valid and useful approach for a review. In their diagnostic role, brokers would test the intent of the question and align it with the policy-makers’ purpose, delineating the perspectives in a way that made their differences clear to the policy team.

Broker 1: “*So, I was just interested in the meaning of that* [question]. *Is it to compare what you do* [with other agencies]*, so literally, ‘What we do, what do other people do?’ Or is there a best practice element to this, ‘What aren’t we doing that we should be doing?’ Or are you specifically wanting to harmonise with what’s happening in other places?*”

The answer would take the reviewers down quite different pathways each looking at the question from a particular angle, for example, a best practice study of survey design, a study of the effectiveness of implementation strategies, or a study of comparative practice, identifying common elements across surveys. While more than one question may feasibly have been answered in the given timeframe, the critical perspective is the one which determines the direction the review will take, namely the logic or line of reasoning against which the search strategy is constructed, the analysis undertaken and the results interpreted.

Policy-makers often found it difficult to pinpoint the critical perspective for themselves. In one review on gendered approaches to interventions for men’s mental health, the policy-makers described the scope of interventions as a continuum from general health and wellbeing to diagnosed mental illness. To narrow the scope for the potential reviewers, the broker had prepared a list of ‘categories’ or groups of men who might be ruled in or out of the review such as returning services personnel and elite athletes. The policy-makers insisted that the review should include all categories and the broker had to explore what they needed in quite a detailed way, before she captured their intent.

Broker 3: “*It sounds like you are talking about help-seeking. So it’s not necessarily people who are diagnosed with clinical depression not adhering to a programme. This seems to be about that first point of contact, and what is a barrier to that, and what helps, and how men recognise it*.”

Policy participant 15: “*Yes, that’s right!*”

The review questions and scope, therefore, took a quite different direction from that prepared by the knowledge broker ahead of the session. Because she was attentive to perspectives on the problem that had not been articulated in the commissioning tool, the broker was able to test these with the policy-makers until the critical perspective was identified and confirmed.

#### Conveying the researcher’s perspective

In their translational role, knowledge brokers explained proposed changes to the wording of review questions, often positioning the change from the perspective of the researchers who would undertake the work, so that the policy-makers could see the review as these researchers might see it.

Broker 3: “*Not because your words are not good, your words are terrific, but they may not be understood so well by a research team*.”

This had a two-fold effect. It explained the reason for the broker’s proposed changes to the questions and scope and situated the changes in the context of the reviewer’s task. It also pointed to the quite different requirements of a research review process, compared to those of a policy process.

Further, brokers explained the proposed changes in terms of their benefit to the policy objectives and in language that was accessible to the policy-makers themselves. One review was designed to clarify the evidence about the effectiveness of a programme that the agency was implementing, which had been challenged by a number of stakeholders. The commissioning team wanted to see how the evidence would align with the components of their programme, and the broker saw a risk in their proposed approach.

Broker 3: “*What we need to do is just turn* [the question] *around so that the reviewer is being asked to do a review of a style of programme, rather than the beginning point being your actual programme and then finding evidence to fit it. Does that make sense? There is a risk of bias there. You want this review to be seen as independent. ‘It’s hands off. We commissioned this review and, independently, this is where the programme stands in relation to the evidence’*.”

It is possible that the policy-makers in this example were not familiar with the question of bias that the broker raised, but they could respond to the advantage of an independent review within a contested environment.

### Task 3: Gaining agreement about content

Policy-makers often found it challenging to narrow the scope of the review, despite a mismatch between what they wanted and the limitations of their timeframe and funding. In addition, some policy participants had a limited understanding of what a research review entailed and therefore of what was possible within a given timeframe. In their diagnostic hat, brokers needed to determine what was feasible within the agency’s constraints and facilitate a process where policy-makers could make judgements about what information would be of greatest value for a particular policy objective.

#### Resolving inconsistencies and enabling decisions

One of the first common tasks brokers encountered was managing diverse perspectives within the policy team about the main focus of the review. While the selection of policy-makers at the session may have been purposeful in terms of the policy task, knowledge brokers could not assume that they were of one mind.

In one review, the policy team had identified a number of issues their policy would address, namely developmental outcomes, school readiness, models of care, workforce roles, technological advances, and cost effectiveness, but time and funding were limited. The broker reflected on what she saw as their central concern quite early in the discussion, but they continued to voice the range of issues. The broker’s approach was to let them ‘talk it out’, allowing them to come to an understanding about the size of the task and the breadth of their perspectives. She was then able to point to what seemed like the two strongest options for the focus of the review, and so narrowed the discussion:

Broker 1: “*So, there are two quite separate directions I think.* [They are] *in the same content area broadly, but there is really something around new and emerging areas; and there is something that is much more around the models of care and implementation.*”

The process was more straightforward when policy-makers had a shared understanding of the focus of the review before the session commenced. The broker’s task then was to confirm rather than negotiate the central focus, ensuring that there was a clear and agreed basis against which decisions were to be made.

In some instances, less experienced brokers seemed to assume that consensus was in place. One broker stated at the beginning of the session that the main focus was clear, but did not check her understanding, possibly assuming that the matter had been agreed ahead of the session. The lack of explicit agreement meant that, at each stage of the session, the different perspectives became apparent and either agreement was sought at each instance, or the senior participant made a decision about what was or was not to be included in the review. This created a situation where decisions at any one point were not aligned to a central focus, and introduced inconsistencies into the questions and scope. Interestingly, one participant raised the question about the review’s main focus, but his fellow participants could not agree. The broker may have resolved any inconsistencies in drafting the proposal, but several points remained unresolved at the close of the session.

#### Weighing up options and fostering judgements

The most common issue brokers negotiated was the choice between two review directions that appeared to be of equal value for the commissioning team, but which together made the review either too large for the available timeframe or funding, or which required distinct kinds of research expertise, so that more than one review team would be needed to answer the questions.

Policy participants did not find this negotiation easy. They were keenly aware of the size and complexity of the policy problem and that narrowing the scope of the review to ensure feasibility inevitably meant that not all issues would be addressed. The knowledge brokers needed to articulate the problem for the policy team and point to potential solutions. One approach was to work with them to prioritise the questions, with a view to excluding those deemed less essential.

Broker 1: “*It may be that you feel you have particular questions around the components of the model and the implementation of it, you know. Are there really important developments around that that you want quite a lot of information about? Or is your greatest concern that there are risk factors that are now well understood that were really only in their infancy then? Because each of these are potentially really quite big questions*.”

A second approach was to prompt the policy team to evaluate which approach would provide best value in their context. This approach required careful management by the brokers who were experts neither in the content area nor in the exigencies of the policy context.

A third approach was to answer the policy questions in a staged way and identify which evidence from research would be needed first. This option focused on the unfolding policy process and the likely timing of the needed information.

Broker 1: “*Sometimes in the past it has actually been more helpful to use a more staged approach. You may already have an idea out of all of these questions what is the most important thing that you need to know now. If I said to you, ‘OK, let’s just say there are three months’, what would you pick as the thing that you most need done in order to inform the activities of your steering group?*”

In developing options, knowledge brokers also drew on approaches beyond a standard review tailored to the needs of the agencies.

Broker 1: “*Another option that is a bit outside the usual model is a slightly different, longer term relationship, where you have got a bit more interaction with one or two key researchers in this area* [over time].”

#### Gaining final agreement

Most brokers concluded the knowledge brokering session by confirming with the policy team the approach that they would take in drafting the review proposal. This was relatively easily done by reviewing the final question set and parameters.

Broker 5: “*Basically, it is going to be four questions: chronic disease; prevention; barriers and factors that affect benefits for different populations; and then strategies to stimulate implementation and adoption: what can we do to make these things work. I think it is a good question set*.”

However, agreement about the questions and scope had not always been reached by the end of the session. In this case, the brokers elected to bring the policy team to an in-principle agreement about the main focus and critical perspective for the review. Consent was then sought for the broker to draft a proposal that was consistent with these minimum parameters. This enabled the brokers to close the session with the central tenets in place so that they were then able to draft a proposal that was feasible and fit for purpose, on the understanding that the wording of the questions and scope might need refinement in a limited, iterative exchange post session.

## Discussion

This study examined knowledge brokers in the context of the Evidence Check rapid review programme, particularly in their diagnostic and translational roles. It offers a novel perspective because it analyses actual instances of interaction between policy-makers and knowledge brokers, identifying and detailing new roles and activities. We anticipate that the findings will be applicable to knowledge brokers with similar models in other contexts.

Overall, the exchange between knowledge brokers and policy-makers in this study is best conceived as an interplay of expertise, where knowledge is pooled and considered in terms of its relevance to the policy process and to the proposed review, permitting a deeper analysis and generating new understandings of policy problems or solutions.

This interplay was reflected in the high level of agreement in the survey between knowledge brokers and policy respondents in terms of the overall value of the knowledge brokering session, changes to the review questions and the perceived value of these changes, and their perception that the information needed to tailor the review for policy purposes had been elicited. In one example, the policy-maker and knowledge broker agreed that the review question had changed in important ways, while also agreeing that the needed information had not been elicited. Free text comments suggested that neither felt they had attained the desired clarity and that both had found the experience somewhat difficult.

Most importantly, knowledge brokers were usually able to gain trust. In their ‘facilitative role’, brokers established trust and respect early in the interaction; this was given form in the shared recognition that the contributions of both knowledge broker and policy-maker were essential to the task. Knowledge brokers trusted policy-makers’ judgement about the policy’s direction and context and policy-makers trusted brokers’ research skills and assessments of feasibility. The mutuality that characterised all sessions is a departure from ‘deficit’ models of knowledge brokering where brokers fill a gap in policy-makers’ research capacity [22, 23, 40, 41] or span an otherwise unbridgeable divide [20, 21, 42].

At the same time, the brokers’ stance in the knowledge brokering session was one of neutrality. While being responsive to the policy team, they did not align themselves with a particular perspective. Their focus was on maximising the potential benefit of a review, allowing policy-makers to consider the strengths and limitations of options, without judgement. It was this neutrality which enabled them to explore participants’ context and options from an independent perspective. Kislov et al. [25] point to the tension brokers can experience in maintaining their ‘in-between’ position and they highlight the ambiguity that can be associated with intermediary roles. In our study, brokers were at once engaged and apart, able to interact with the policy team, remain neutral in terms of the selection of options, and be purposeful in ensuring a review that was both feasible and matched to policy-makers’ needs.

The ‘diagnostic role’ enacted by the knowledge brokers in this study goes beyond having a generic familiarity with policy processes and environments [25]. Discourse about the need to be conscious of the policy context to achieve a shared understanding about an agency’s needs [19, 23, 24] also falls short of capturing the kind of nuanced information that reviewers require [3, 43]; in any given context, some factors will be more salient to the policy process than others [44]. The brokers’ task is to work with the agency to refine the review questions and scope so that they take into account parallel policy activity and constraints in such a way that the review captures precisely what is needed [34]. Unpacking the relationship between contextual factors, review questions and the policy process is therefore a critical function for knowledge brokers.

Knowledge brokers had a nuanced understanding of the ways in which meaning is constructed in policy and in research discourse. In their ‘interpretative role’, brokers paid close attention to the ways in which words and phrases were used, pinpointing with precision those that would be difficult to interpret for the researchers and defining and fine-tuning these as the sessions evolved. They drew on their knowledge about the way research is accessed and organised and their experience and understanding about how review questions are best framed and structured.

The critical act of knowledge translation in this study lay in the movement from the policy perspective, to the reviewers’ perspective, and again to the policy perspective; this shift in perspectives was inherent in all sessions. Knowledge brokers checked that they had understood what policy participants intended, tested this against the logic of the research process, and confirmed that their research construct was consistent with policy-makers’ intent. Policy-makers tended to hold to their central conviction about what the review should address but moved with the broker through alternative constructs until a shared understanding was achieved.

This highlights a ‘deliberative role’ for knowledge brokers. Deliberation as a concept has emerged in response to the need for a considered exploration of the ways in which knowledge from research can or should contribute to policy-making, taking into account features of the local environments such as structures, resources, interests, alliances, ideologies and the potential roles of stakeholders [29, 30]. Deliberative processes have the potential to influence decision-making at local and at organisational levels [11, 45] and can increase the relative weight of research in decision-making [46]. In our study, the deliberative function took the form of a ‘looking forward’ to anticipate how the potential findings from a review might inform a policy process or interact with contextual factors to bring about change – a preview against which choices of review focus might be made, but many other applications will be possible. The deliberative function of knowledge brokers is also likely to be relevant to other settings where there are multiple perspectives on a policy problem or solution, where there are contested policy environments or where these perspectives need to be evaluated against a particular policy objective.

The facilitative, diagnostic, interpretative and deliberative roles identified in this study add considerable depth to those reported by Bornbaum et al. [33] in their systematic review of knowledge brokering, namely of knowledge management, linkage and exchange, and capacity-building. The authors describe the multiple activities associated with these roles; however, the complex interactions between policy-makers, the policy process and the policy or political environments were not highlighted. The roles identified in this study draw attention to the underlying processes knowledge brokers used, such as exploring complex interactions, establishing the relationships between factors and their relative importance to the review question, and testing and reframing their understanding in conventions of both policy and research.

The skill complexity highlighted here may provide guidance for the selection and training of knowledge brokers. Knowledge brokers must have the kind of credibility that engenders trust [39], understand the ways in which research is used and have the capacity to explore sensitive policy contexts in a neutral way. Further, it would be difficult for a knowledge broker to undertake this work without being familiar with policy contexts and with the complexities likely to exist in policy processes. This suggests that the role may be more suited to senior researchers with some policy experience. Knowledge broker training may usefully include understanding the perspectives from which a policy problem may be viewed, reframing these from the perspective of potential reviewers, communicating research concepts in policy-friendly language and evaluating the potential benefits of options against policy objectives.

While the number of knowledge brokering sessions conducted was used in this study as an indicator of experience, what actually constitutes expertise in knowledge brokering is rather more complex as outlined above. Nevertheless, some knowledge brokers demonstrated greater skill complexity than others, showing an ability to sift dense narratives, identifying issues that would be pertinent to decisions about the review’s focus and direction and leaving aside others; linguistic and conceptual expertise to frame and reframe both policy and research concepts in the language of the participants; a genuine neutrality that enables policy participants to actively critique what they want from a review and how this can best be achieved; a sensitivity to meaning and intention and to the ways in which these are signalled; and eliciting and bringing together policy-makers’ expertise in clarifying and defining problems and solutions.

Taken together, these findings point to a knowledge brokering process that is simultaneously ‘translational’, in that it moves between two disciplines with quite different conceptual and linguistic conventions and aligns them, reframing both policy and research constructs in the language of the policy participants; ‘diagnostic’, in that the broker seeks detailed information from the policy participants and hones this recursively throughout the session, refining the scope to reflect this information; ‘deliberative’, in that policy participants are not simply providing information, but are actively critiquing and revising what they want from the review and how this can be achieved; ‘facilitative’ in that the broker is highly sensitised to cues about meaning, intention, expectations and understandings, and responds to these cues, adjusting their technique to facilitate trust and maximise progress.

Overall, the knowledge of both brokers and policy-makers is combined to generate new perspectives on the policy problem and to open and contextualise a field of enquiry, extending policy-makers’ understanding of what is possible from research and what is feasible within the constraints of a rapid review.

Future research might usefully examine the potential relationship between the use of knowledge brokering at the commencement of a rapid review process and the extent to which they are used by the policy-makers who commission them. It would also be of benefit to understand whether other rapid review programmes using knowledge brokers have had similar experiences, and to examine the transferability of the four knowledge brokering roles to other contexts and settings.

## Conclusion

This study identified four key roles for knowledge brokers, namely facilitative, diagnostic, interpretative and deliberative roles. Knowledge brokers were able to establish rapport, enable disclosure of essential information and explore contextual factors, and link these to the review’s purpose and intended use. These brokers were trusted and neutral intermediaries whose knowledge of research and policy contexts enabled them to move easily between policy and research perspectives, assist policy-makers to evaluate various options and craft a review proposal that was targeted, responsive and feasible. Mutuality, respect and an interplay of expertise were integral to the knowledge brokering process. Future research might usefully examine the transferability of the four knowledge brokering roles to other contexts and settings.

## Box 1 Examples of knowledge broker and policy-makers’ free text responses

**EXAMPLE 1**

**Knowledge broker**

• “[I have a] *better understanding of the relative importance of the questions and a better understanding of the practical context in which the review findings will be used. There were differences in focus amongst the agency representatives (micro vs. bigger picture focus) but there was overall concordance about the purpose of the rapid review*.”

**Policy participants**

• “*Clarification of the questions, scope: these were not major changes, but the questioning allowed us to be more specific about our needs and to flag some additional information we could provide as background*.”

• “*It really helped us to clarify what we wanted and to emphasise that whatever* [the reviewers] *would find would be really useful. I think that* [the broker] *gained a better understanding of our requirements, which is actually a relief. It fills me with more confidence that the* [reviewers] *will provide us with what we are after*.”

**EXAMPLE 2**

**Knowledge broker**

• “[I have] *a better understanding of the dynamics of the group involved in the session. It was apparent that there was no group consensus about what was required from the outcomes of a review. This was a difficult session characterised by a limited comprehension of what a rapid review can and can’t deliver*.”

**Policy participant**

• “*I’m not sure the process seemed to clarify things as I expected. The knowledge broker still seems to think that the required review is of a greater magnitude than I or my colleagues do. It makes me think that the needs are actually still very unclear. I need to put more thought into exactly what we are asking for, as after the session it felt that things were very ‘woolly’*.”

**EXAMPLE 3**

**Knowledge broker**

• “*The agency appreciation and understanding of the nature, value and process of an independent evidence check* [changed]. [There was a] *broadening of agency focus about the reason for the Evidence Check, i.e. not focused on ‘is our programme evidence based?’ A very pleasant and constructive group to work with, who engaged well with commissioning tool. It really makes a positive difference when the agency is more interested in doing something thoroughly rather than quickly!*”

**Policy participants**

• “*I feel that the brokering session allowed the development of a more detailed and research focussed approach. As someone with limited research experience, our questions had possibly lacked consideration of the practical process of undertaking research. The knowledge broker’s input strengthened the independence of the proposal by removing some of the inherent bias of the questions towards our expected outcomes*.”

• “*The knowledge broker’s external opinion allowed the research questions to be viewed in a slightly different way. I felt that the knowledge broker was friendly and approachable, and therefore I felt comfortable in challenging some of her ideas where they didn’t fit with our needs and accepting others*.”

## Box 2 Knowledge broker activities

Knowledge brokers elicited information by using a range of types of question (open, closed, hypothetical), drawing out more detail, using examples to clarify meaning and checking the definition of key terms. Careful management of the session ensured that information needed by the brokers was captured and their understanding of what was required was tested, refined and confirmed.

Knowledge brokers explored the underlying contextual factors by creating conditions that enabled an open dialogue, coming to a shared understanding of the policy environment (including sensitivities that could not be shared with the researchers), being attentive to the range of viewpoints from which a problem could be articulated and evaluating contextual information in light of the policy objectives.

Knowledge brokers negotiated options for the content of a review by aligning them with the policy purpose and context, identifying critical perspectives, highlighting inconsistencies, articulating alternative review question sets and confirming agreement of the final review parameters.

## Additional file

Additional file 1: Knowledge broker and participant surveys. (DOCX 93 kb)

## References

[1] Innvær S, Vist G, Trommald M, Oxman A (2002). Health policy-makers perceptions of their use of evidence: a systematic review. J Health Serv Res Policy.

[2] Oliver K, Innvar S, Lorenc T, Woodman J, Thomas J (2014). A systematic review of barriers to and facilitators of the use of evidence by policymakers. BMC Health Serv Res.

[3] Campbell D, Donald B, Moore G, Frew D (2011). Evidence Check: knowledge brokering to commission research reviews for policy. Evid Policy.

[4] Brennan SE, Cumpston M, Misso ML, McDonald S, Murphy MJ (2016). Design and formative evaluation of the Policy Liaison Initiative: a long-term knowledge translation strategy to encourage and support the use of Cochrane systematic reviews for informing health policy. Evid Policy.

[5] Brownson RC, Dodson EA, Stamatakis KA, Casey CM, Elliott MB (2011). Communicating evidence-based information on cancer prevention to state-level policy makers. J Natl Cancer Inst.

[6] van der Heide I, van der Noordt M, Proper KI, Schoemaker C, van den Berg M (2016). Implementation of a tool to enhance evidence-informed decision making in public health: identifying barriers and facilitating factors. Evid Policy.

[7] Dilworth K, Tao M, Shapiro S, Timmings C (2013). Making health promotion evidenced-informed: an organizational priority. Health Promot Pract.

[8] Jansen MW, Hoeijmakers M (2013). A masterclass to teach public health professionals to conduct practice-based research to promote evidence-based practice: a case study from the Netherlands. J Public Health Manag Pract.

[9] Yost J, Ciliska D, Dobbins M (2014). Evaluating the impact of an intensive education workshop on evidence-informed decision making knowledge, skills, and behaviours: a mixed methods study. BMC Med Educ.

[10] Morris ZS, Bullock A, Atwell C (2013). Developing engagement, linkage and exchange between health services managers and researchers: Experience from the UK. J Health Serv Res Policy.

[11] Moat KA, Lavis JN, Clancy SJ, El-Jardali F, Pantoja T. Evidence briefs and deliberative dialogues: perceptions and intentions to act on what was learnt. Bull World Health Organ. 2013;92:20–8 10.2471/BLT.12.116806 PMC3865546 24391297

[12] Bullock A, Morris ZS, Atwell C (2012). Collaboration between health services managers and researchers: making a difference?. J Health Serv Res Policy.

[13] Wathen CN, Sibbald SL, Jack SM, MacMillan HL (2011). Talk, trust and time: a longitudinal study evaluating knowledge translation and exchange processes for research on violence against women. Implement Sci.

[14] Heaton J, Day J, Britten N (2016). Collaborative research and the co-production of knowledge for practice: an illustrative case study. Implement Sci.

[15] Rycroft-Malone J, Burton CR, Bucknall T, Graham ID, Hutchinson AM (2016). Collaboration and co-production of knowledge in healthcare: opportunities and challenges. Int J Health Policy Manag.

[16] Lomas J (2007). The in-between world of knowledge brokering. BMJ.

[17] Conklin J, Lusk E, Harris M, Stolee P (2013). Knowledge brokers in a knowledge network: the case of Seniors Health Research Transfer Network knowledge brokers. Implement Sci.

[18] Dagenais C, Laurendeau M-C, Briand-Lamarche M (2015). Knowledge brokering in public health: a critical analysis of the results of a qualitative evaluation. Eval Program Plann.

[19] Ward V, House A, Hamer S (2009). Knowledge brokering: the missing link in the evidence to action chain?. Evid Policy.

[20] Hammami H, Amara N, Landry R (2013). Organizational climate and its influence on brokers knowledge transfer activities: a structural equation modeling. IJIM.

[21] Long JC, Cunningham FC, Braithwaite J (2013). Bridges, brokers and boundary spanners in collaborative networks: a systematic review. BMC Health Serv Res.

[22] Dobbins M, Robeson P, Ciliska D, Hanna S, Cameron R (2009). A description of a knowledge broker role implemented as part of a randomized controlled trial evaluating three knowledge translation strategies. Implement Sci.

[23] Traynor R, DeCorby K, Dobbins M (2014). Knowledge brokering in public health: a tale of two studies. Public Health.

[24] Robeson P, Dobbins M, DeCorby K (2008). Life as a knowledge broker in public health. JCHLA/JABSC.

[25] Kislov R, Wilson P, Boaden R (2017). The dark side of knowledge brokering. J Health Serv Res Policy.

[26] Elueze IN (2015). Evaluating the effectiveness of knowledge brokering in health research: a systematised review with some bibliometric information. Health Inf Libr J.

[27] Haynes AS, Gillespie JA, Derrick GE, Hall WD, Redman S (2011). Galvanizers, guides, champions, and shields: the many ways that policymakers use public health researchers. Milbank Q.

[28] CIPHER Investigators (2014). Supporting Policy In health with Research: an Intervention Trial (SPIRIT)-protocol for a stepped wedge trial. BMJ Open.

[29] Muntaner C, Chung H, Murphy K, Ng E (2012). Barriers to knowledge production, knowledge translation, and urban health policy change: ideological, economic, and political considerations. J Urban Health.

[30] Head BW (2008). Three lenses of evidence-based policy. Aust J Publ Adm.

[31] Lavis JN, Permanand G, Lavis JN, Catallo C (2014). A way to approach knowledge brokering: the BRIDGE framework and criteria. Chapter 2. Bridging the Worlds of Research and Policy in European Health Systems.

[32] Dobbins M, Hanna SE, Ciliska D, Manske S, Cameron R (2009). A randomized controlled trial evaluating the impact of knowledge translation and exchange strategies. Implement Sci.

[33] Bornbaum CC, Kornas K, Peirson L, Rosella LC (2015). Exploring the function and effectiveness of knowledge brokers as facilitators of knowledge translation in health-related settings: a systematic review and thematic analysis. Implement Sci.

[34] Moore G, Redman S, D’Este C, Makkar S, Turner T (2017). Does knowledge brokering improve the quality of rapid review proposals? A before and after study. Syst Rev.

[35] Moore G, Redman S, Rudge S, Haynes A (2018). Do policy-makers find commissioned rapid reviews useful?. Health Res Policy Syst.

[36] The Sax Institute. Evidence Check Program. 2018. https://www.saxinstitute.org.au/our-work/knowledge-exchange/evidence-check/. [Accessed 27 Mar 2018]

[37] Guest G, Bunce A, Johnson L (2006). How many interviews are enough? An experiment with data saturation and variability. Field Methods.

[38] Charmaz K (2014). Constructing Grounded Theory.

[39] Haynes AS, Derrick GE, Redman S, Hall WD, Gillespie JA (2012). Identifying trustworthy experts: how do policymakers find and assess public health researchers worth consulting or collaborating with?. PLoS One.

[40] Ward M, Dobbins M, Peirson L (2016). Lessons learnt from implementing an organizational strategy for evidence-informed decision making. Public Health Panor.

[41] Armstrong R, Waters E, Dobbins M, Anderson L, Moore L (2013). Knowledge translation strategies to improve the use of evidence in public health decision making in local government: intervention design and implementation plan. Implement Sci.

[42] Knight C, Lyall C (2013). Knowledge brokers: the role of intermediaries in producing research impact. Evid Policy.

[43] Dwan KM, McInnes PC (2013). Increasing the influence of ones research on policy. Aust Health Rev.

[44] Moat KA, Lavis JN, Abelson J (2013). How contexts and issues influence the use of policy-relevant research syntheses: a critical interpretive synthesis. Milbank Q.

[45] Boyko JA, Lavis JN, Abelson J, Dobbins M, Carter N (2012). Deliberative dialogues as a mechanism for knowledge translation and exchange in health systems decision-making. Soc Sci Med.

[46] Flitcroft K, Gillespie J, Salkeld G, Carter S, Trevena L (2011). Getting evidence into policy: the need for deliberative strategies?. Soc Sci Med.

