# Supplementary material for: Deconstructing knowledge brokering for commissioned rapid reviews: an observational study
Source: Health Res Policy Syst. 2018 Dec 12;16:120. doi: 10.1186/s12961-018-0389-7 (PMC6292028; doi:10.1186/s12961-018-0389-7)
Supplement: Supplementary file 1 — Knowledge broker and participant surveys. (DOCX 93 kb) [file 12961_2018_389_MOESM1_ESM.docx]

**Additional file 1: Knowledge broker and participant surveys**

KBS01 PMV01

**‘KNOWLEDGE BROKERS: WHAT DO THEY DO AND HOW DO THEY DO IT?’**

**BRIEF SURVEY FOR POLICYMAKERS AND PROGRAM MANAGERS**

1. What do you think changed, if anything, during the knowledge brokering session?

­­­­­­­­­­­­­­­­­­­­­­­­­________________________________________________________________________

________________________________________________________________________

________________________________________________________________________

________________________________________________________________________

1. Did the knowledge brokering session change your understanding of the policy problem?

*Please* ***circle or highlight in bold*** *the one response which most closely reflects your view*

| ☐ Yes a lot | ☐ Yes a little | ☐ No, not at all | ☐ Don’t know |
| --- | --- | --- | --- |

1. Was this change in understanding important?

*Please* ***circle or highlight in bold*** *the one response which most closely reflects your view*

| ☐ Yes a lot | ☐ Yes a little | ☐ No, not at all | ☐ Don’t know |
| --- | --- | --- | --- |

1. Did the knowledge brokering session change the way the research question was specified?

*Please* ***circle or highlight*** *in bold the one response which most closely reflects your view*

| ☐ Yes a lot | ☐ Yes a little | ☐ No, not at all | ☐ Don’t know |
| --- | --- | --- | --- |

1. Was this change in research question important?

*Please* ***circle or highlight*** *in bold the one response which most closely reflects your view*

| ☐ Yes very | ☐ Yes a little | ☐ No, not at all | ☐ Don’t know |
| --- | --- | --- | --- |

1. Did the information elicited during the session help target the review to your needs?

*Please* ***circle or highlight*** *in bold the one response which most closely reflects your view*

| ☐ Yes a lot | ☐ Yes a little | ☐ No, not at all | ☐ Don’t know |
| --- | --- | --- | --- |

1. Overall did you find the knowledge brokering session valuable?

*Please* ***circle or highlight*** *in bold the one response which most closely reflects your view*

| ☐ Yes a lot | ☐ Yes a little | ☐ No, not at all | ☐ Don’t know |
| --- | --- | --- | --- |

1. Is there anything else you would like to tell us about your experience as a participant in today’s knowledge brokering session?

_______________________________________________________________________

_______________________________________________________________________

_______________________________________________________________________

_______________________________________________________________________

_______________________________________________________________________

_______________________________________________________________________

KBS01 KBV01

**‘KNOWLEDGE BROKERS: WHAT DO THEY DO AND HOW DO THEY DO IT?’**

**BRIEF SURVEY FOR KNOWLEDGE BROKERS**

1. What do you think changed, if anything, during the knowledge brokering session?

­­­­­­­­­­­­­­­­­­­­­­­­­________________________________________________________________________

________________________________________________________________________

________________________________________________________________________

________________________________________________________________________

1. Did the knowledge brokering session change your understanding of the policy problem?

*Please* ***circle or highlight in bold*** *the one response which most closely reflects your view*

| ☐ Yes a lot | ☐ Yes a little | ☐ No, not at all | ☐ Don’t know |
| --- | --- | --- | --- |

1. Do you think this change in understanding was important?

*Please* ***circle or highlight in bold*** *the one response which most closely reflects your view*

| ☐ Yes a lot | ☐ Yes a little | ☐ No, not at all | ☐ Don’t know |
| --- | --- | --- | --- |

1. Did the knowledge brokering session change the way the research question was specified?

*Please* ***circle or highlight*** *in bold the one response which most closely reflects your view*

| ☐ Yes a lot | ☐ Yes a little | ☐ No, not at all | ☐ Don’t know |
| --- | --- | --- | --- |

1. Was this change in research question important?

*Please* ***circle or highlight*** *in bold the one response which most closely reflects your view*

| ☐ Yes a lot | ☐ Yes a little | ☐ No, not at all | ☐ Don’t know |
| --- | --- | --- | --- |

1. Was the information you needed to target the review to the policy maker’s needs, provided in today’s session?

*Please* ***circle or highlight*** *in bold the one response which most closely reflects your view*

| ☐ Yes a lot | ☐ Yes a little | ☐ No. not at all | ☐ Don’t know |
| --- | --- | --- | --- |

1. Overall did you find the knowledge brokering session valuable?

*Please* ***circle or highlight*** *in bold the one response which most closely reflects your view*

| ☐ Yes a lot | ☐ Yes a little | ☐ No, not at all | ☐ Don’t know |
| --- | --- | --- | --- |

KBS01 KBV01

1. Is there anything else you would like to tell us about your experience as a knowledge broker in today’s session?

­­­­­­­­­­­­­­­­­­­­­­­­­________________________________________________________________________

________________________________________________________________________

________________________________________________________________________

________________________________________________________________________

________________________________________________________________________
